# Prescription Opioid Use among Patients with Chronic Noncancer Pain before and after the COVID-19 Outbreak in Taiwan: A Multicenter Prospective Observational Study

**DOI:** 10.3390/healthcare10122460

**Published:** 2022-12-06

**Authors:** Jia-Lin Chen, Shung-Tai Ho, Wei-Zen Sun, Yu-Chuan Tsai, Kuang-I Cheng, Yen-Chin Liu, Yi-Jer Hsieh, Yeong-Ray Wen, Po-Kai Wang, Chun-Sung Sung, Chun-Chang Yeh, Tso-Chou Lin

**Affiliations:** 1 Department of Anesthesiology, Tri-Service General Hospital, National Defense Medical Center, Taipei 11490, Taiwan; 2 Department of Anesthesiology, Kaohsiung Medical University Hospital, Kaohsiung Medical University, Kaohsiung 80756, Taiwan; 3 Department of Anesthesiology, National Taiwan University Hospital, National Taiwan University, Taipei 10617, Taiwan; 4 Health Science & Wellness Center, National Taiwan University, Taipei 10617, Taiwan; 5 Department of Anesthesiology, Center of Pain Management, E-Da Cancer Hospital, School of Medicine, I-Shou University College of Medicine, Kaohsiung 82445, Taiwan; 6 Department of Anesthesiology, School of Post-Baccalaureate, College of Medicine, Kaohsiung Medical University, Kaohsiung 80756, Taiwan; 7 Department of Anesthesiology, National Cheng Kung University Hospital, College of Medicine, National Cheng Kung University, Tainan 70403, Taiwan; 8 Department of Anesthesiology, Changhua Christian Hospital, Changhua 50006, Taiwan; 9 Department of Anesthesiology, China Medical University Hospital, China Medical University, Taichung 40447, Taiwan; 10 Department of Anesthesiology, Hualien Tzu Chi Hospital, Buddhist Tzu Chi Medical Foundation, School of Medicine, Tzu Chi University, Hualien 97004, Taiwan; 11 Department of Anesthesiology, Taipei Veterans General Hospital, Taipei 11217, Taiwan

**Keywords:** chronic pain, noncancer, opioid, prescription, COVID-19

## Abstract

Background: The COVID-19 outbreak disrupted medical access for patients receiving chronic opioid therapy. This study investigated their prescription opioid dosages before and after the 2020 outbreak in Taiwan. Methods: A prospective questionnaire survey was conducted among registered outpatients receiving long-term opioids before July 2019 in Taiwan. The questionnaire included items from the Taiwanese Brief Pain Inventory and quality of life assessment. Follow-up surveys in outpatient departments through October 2020 were conducted to collect opioid prescription data. Results: After a mean of 531 days, the questionnaire responses of 103 of the initial 117 respondents were reviewed. Daily opioid doses decreased for 31 respondents (30.1%), remained roughly equivalent (defined as ±2.5%) for 27 (26.2%), and increased for 45 (43.7%) after the first wave of the pandemic. The use of strong opioids and nonopioid medications did not significantly differ among the three groups, but less fentanyl patch use was noted in the decreased-dose group after the outbreak. More than 70% of the patients received daily high-dose opioids (≥90 morphine milligram equivalents); moreover, 60% reported constipation. No deaths due to opioid overdose occurred during the study period. Conclusions: The COVID-19 outbreak in 2020 did not interrupt access to long-term opioid prescriptions for most registered patients with chronic pain in Taiwan. Less fentanyl patch use was observed in participants whose opioid dose was tapering.

## 1. Introduction

The United States and Canada have faced an opioid crisis for the past two decades. The 2011 Canadian guidelines for safe opioid use defined a “watchful” daily dose of 200 morphine milligram equivalents (MMEs) as requiring careful reassessment [1]. In 2016, the US Centers for Disease Control and Prevention (CDC) revised the high-dose threshold to 90 MMEs/day in the *CDC Guideline for Prescribing Opioids for Chronic Pain* [2]. After tremendous efforts to reduce opioid prescriptions for acute and chronic pain in the United States, the rate of national overdose deaths due to prescription opioids gradually declined from 36% in 2017 to 32% in 2018 [3], 20% in 2019, and 17.9% in 2020 [4]. In February 2022, the CDC website posted a draft of the *CDC Clinical Practice Guideline for Prescribing Opioids—United States, 2022* and the release of the final updated guideline is anticipated in late 2022 [5].

Since March 2020, the COVID-19 pandemic and associated lockdowns have increased morbidity risks, mortality risks, and healthcare utilization among patients with chronic pain [6], as well as the overuse of opioids as substitutes for physical and psychological treatments [7] and opioid overdose-related emergency department visits [8]. Nevertheless, rapid tapering or abrupt discontinuation of opioids may pose new risks, namely opioid withdrawal [9] and suicidal ideation [10], especially among patients coming off of long-term high-dose opioid therapy [11]. Additionally, patients discharged after COVID-19 hospitalization may encounter further cognitive, physical, and psychological dysfunction causing profound effects on quality of life [12,13]. Clinical and public health resources should be reallocated to ensure the continuity of pain medication prescription and biopsychosocial management, including telemedicine, during crisis periods [14].

Following the Anglo–Sino Opium War in the 1840s, the use of opioids had been prohibited in China. Until 2011, more than 70% of Taiwanese physicians perceived physician reluctance as a leading barrier to prescribing opioids for chronic pain [15]. In Taiwan, the prescription of long-term opioids for the management of chronic noncancer pain (CNCP) has been strictly regulated since 1996 [16]. In January 2022, the Taiwan Food and Drug Administration adjusted the recommended daily opioid dose from 200 to 100 MMEs for most patients [16]. Accordingly, opioid therapy and psychiatric evaluations must be regularly audited by the Narcotics Management Committee at a medical center or regional hospital but not by physicians at local clinics. Moreover, the treating hospital should submit a report on opioid therapy and patient evaluation to the Taiwan Food and Drug Administration for surveillance at least every 4 months. Each patient with CNCP should obtain a long-term opioid prescription from only one physician, and those with aberrant behaviors suggestive of opioid misuse must be reported to the hospital’s opioid committee for the discontinuation of opioid treatment [16]. Two studies using official patient lists from the Taiwan Food and Drug Administration have reported that only 114 and 328 registered patients with CNCP received chronic opioid therapy in 2001 and 2010, respectively [17,18]. Therefore, the prescription of long-term opioids for managing CNCP and the subsequent opioid crisis are extremely rare in Taiwan, but widespread in the United States.

The first COVID-19 outbreak in Taiwan was well controlled. There were only 447 laboratory-confirmed cases before 30 June 2020, [19] and 873 cases by the end of December 2020 in a population of over 23 million [20]. However, restricted hospital entry and exposure concerns may have limited medical service access for outpatients with CNCP during this period. In 2019, we conducted a cross-sectional survey to examine sex differences in the prevalence of depression and suicidal ideation and sex hormones among Taiwanese patients with CNCP [21], including a prospective follow-up questionnaire survey and assessment of opioid prescription use in 2020. In this secondary study, we analyzed the doses of prescription opioids before and after the first wave of the COVID-19 pandemic (i.e., 2019 vs. 2020) in Taiwan. In addition, we investigated the physical and psychological effects of the use of high-dose opioids.

## 2. Materials and Methods

### 2.1. Participants

A prospective multicenter questionnaire survey was initially administered to registered outpatients receiving long-term opioids for CNCP in Taiwan between October 2018 and July 2019 [21]. A “long-term” prescription refers to the continuous use of a drug for more than 14 days or intermittent use exceeding 28 days within 3 months [16]. A trained research assistant visited the outpatient departments of nine medical centers in Taiwan, and pain medicine specialists identified registered patients with CNCP to determine their interest in this study. Patients who could not read or write were excluded. Participants completed the questionnaire by themselves or with verbal help from the research assistant. All participants provided written informed consent, and this study received ethics approval from the relevant institutional review boards of nine medical centers (TSGHIRB-2-106-05-162, NTUH-201810037RINC, TVGHIRB-2018-12-010BC, CMUH-108-REC2-029, CCHIRB-181118, NCKUHIRB-B-ER-108-008, KMUHIRBE II-20190028, EDAHIRB-EMRP-107-118, and HTCH-IRB107-212-B).

### 2.2. Study Instrument

This study used a Chinese-language questionnaire incorporating elements from several existing inventories [18,21,22], namely the Taiwanese version of the Brief Pain Inventory [23], the Chinese version of the Beck Depression Inventory-II, and the Taiwanese version of the abbreviated World Health Organization Quality of Life (WHOQOL-BREF) questionnaire [24]. The initial questionnaire investigated chronic pain duration, opioid therapy duration, pain intensity (scored from 0 to 10, with 0 indicating the least pain and 10 the worst pain), and pain reduction percentage (0–100%) after receiving opioids in the previous week. In addition, the questionnaire investigated adverse effects (e.g., constipation) and pain-related interference (scored from 0 to 10, with 0 indicating the least interference and 10 the worst interference) with daily function, including general activities, mood, ability to walk, normal work activities, relationships with others, sleep, and enjoyment of life, before and after receiving opioids in the previous week [23]. We also examined depressive condition in the past week and aberrant behaviors associated with opioid prescription misuse in the past month, including the frequency of borrowing pain medication from someone else and visiting the emergency room for more pain medication. The WHO Quality of Life scores of physical, psychological, social, and environmental domains are summarized as a total score of four domains (ranging from 16 to 80) [24]. Pain medicine specialists in outpatient departments provided the medication lists of opioid and nonopioid prescriptions before and after the COVID-19 outbreak in 2020. Daily oral MME values were calculated using the following conversion factors [21]: 3 for intramuscular morphine, 0.15 for codeine, 0.1 for oral meperidine, 0.4 for intramuscular meperidine, 1.5 for oxycodone, 0.1 for tramadol, 2.4 for transdermal fentanyl (mcg/hour) [2], 40 for sublingual buprenorphine, and 2 for and transdermal buprenorphine [1,2,25].

### 2.3. Follow-Up Survey on Opioid Prescriptions

A follow-up survey was scheduled to be conducted 1 year after the initial questionnaire investigation; however, it was postponed because of the COVID-19 outbreak in January 2020. Because of restricted hospital entry and exposure concerns in Taiwan, the follow-up survey was truncated to the collection of opioid prescription data by pain medicine specialists in the outpatient departments, continuing through October 2020, after the first wave of the pandemic. The doses at follow-up were compared with the corresponding prepandemic doses, and the patients were arbitrarily divided into three groups consisting of those receiving a decreased daily dose (greater than 2.5%), an equal daily dose (±2.5%), or an increased daily dose (greater than 2.5%). The threshold for a “high” daily dose was defined as ≥200, ≥90, or ≥100 MMEs on the basis of the 2011 Canadian guideline [1], 2016 CDC guideline [2], or 2022 Taiwan physician guideline [16], respectively. The associated physical and psychological effects of the three groups were also analyzed.

### 2.4. Statistical Analysis

Demographic data are presented as means ± standard deviations. Questionnaire responses were analyzed using SPSS version 22 (IBM, Armonk, NY, USA). The Kruskal–Wallis one-way analysis of variance (ANOVA) and Mann–Whitney *U* test were used to compare opioid and nonopioid medications, side effects, depression scores, and quality of life scores among the groups (increased, decreased, or equal daily dose). Categorical variables were compared using the chi-squared test or Fisher’s exact test. In all cases, a *p* value of <0.05 was considered statistically significant.

## 3. Results

**Figure 1:**
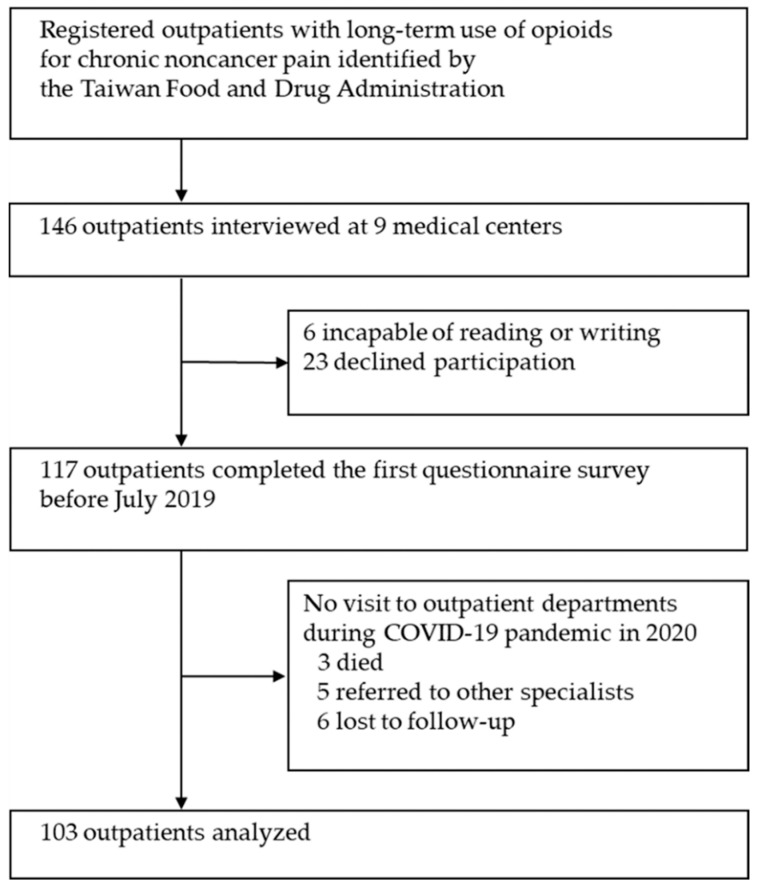
Participant enrollment flowchart.

Of the 117 patients who responded to the first questionnaire in 2019, 103 (88.0%) were reviewed at the outpatient departments in the follow-up survey after the COVID-19 outbreak in 2020. Fourteen (12.0%) patients were excluded; three died of underlying diseases (not opioid overdose), five were referred to other specialists, and six were lost to follow-up (Figure 1).

Table 1 presents patient data. The average follow-up duration was 531.0 ± 57.6 days, and the median prepandemic durations of chronic pain and opioid therapy were 126 and 87 months, respectively. Among the 103 patients (40 women and 63 men) who completed the survey after the outbreak period, 31 (30.1%) received decreased daily doses (including one patient who discontinued opioid use), 27 (26.2%) equal doses (±2.5%), and 45 (43.7%) received increased doses. The leading five diagnoses were failed back surgery syndrome, chronic pancreatitis, neuralgia, spinal cord injury, and spondylosis with radiculopathy in 23 (22.3%), 15 (14.6%), 15 (14.6%), 13 (12.6%), and 8 (7.8%) patients, respectively, amounting to 74 (71.8%) of the 103 patients. The ratio of married to single status was significantly lower in the increased-dose group (*p* = 0.010) than in the other two groups.

Table 2 reveals that a median reduction of 60 mg/day (46.7%) was observed in the decreased-dose group; the median escalation in the increased-dose group was 55.7 mg/day (40%). The total daily opioid dose across all the groups increased slightly, from 21,789 MMEs before the outbreak to 22,240 MMEs (+2.1%) after the outbreak. On the basis of the US (90 MMEs) and Taiwanese (100 MMEs) thresholds, 73 (70.8%) and 70 (68.0%) patients, respectively, received high-dose opioids. No differences were observed regarding strong opioid prescriptions or nonopioid medications among the three groups except for a reduction in fentanyl patch use in the decreased-dose group (*p* = 0.013; from five patients before the outbreak to one patient receiving 28.8 MMEs after the outbreak).

As indicated in Table 3, the most common side effect was constipation, reported by 62 (60.2%) of 103 patients before the first COVID-19 outbreak. However, only 28 (27.2%) and 35 (34.0%) patients received laxatives before and after the outbreak, respectively. All patients had low scores for global health (2.41 ± 0.89, max 5), and overall quality of life (2.05 ± 0.84, max 5), as well as low scores for the four domains of physical health, psychological health, social relationships, and environment (transformed score 41.96 ± 10.99, max 80). The depression, suicidal ideation, and quality of life scores of the decreased-dose group were not significantly different from the scores of the equal-dose and increased-dose groups.

As presented in Table 4, only 33 (32.0%) of the 103 patients were concerned about opioid addiction, and 61 (59.2%) were unwilling to stop opioid treatment. Nearly 5% of patients reported frequently taking more medication than prescribed, without significant differences among the three groups. Only one or two patients in each group had frequently visited the emergency department for additional pain medication during the past month, but more than 20% of patients in the decreased-dose and increased-dose groups occasionally visited the emergency department for additional pain medication (*p* = 0.010 and 0.002, respectively); in contrast, zero patients did so in the equal-dose group.

## 4. Discussion

### 4.1. Major Findings

To the best of our knowledge, this is the first prospective longitudinal study examining long-term opioid prescriptions among registered patients with CNCP in Taiwan before and after the COVID-19 outbreak. Of the 103 patients surveyed after the first wave of the pandemic, 30% had a median reduction of 60 MMEs, whereas 43.7% had an increase of 55.7 MMEs. We observed decreased usage of the fentanyl patch in the decreased-dose group after the outbreak. However, we noted no other differences in the use of other strong opioids or nonopioid medications between the decreased-dose group and the equal-dose and increased-dose groups. Daily high-dose opioids (≥90 MMEs) were prescribed to 70% of all the patients after the outbreak in 2020.

### 4.2. Impact of COVID-19 Outbreak and Opioid Overdose

Multimodal therapy is optimal for managing chronic pain. However, pharmacological, physical, and psychological treatments for chronic pain were disrupted by the COVID-19 pandemic. In the Chronic Pain & COVID-19 Pan-Canadian Study, 38.3% and 68.3% of patients reported changes in their pharmacological pain treatment and self-management strategies, respectively, during the early pandemic period of 2020 [26]. Lack of access to medication during lockdown, enforced isolation, and cessation of physical and psychological treatments, combined with social and economic stressors, may have increased compensatory opioid intake by patients with prescriptions [7]. Additionally, physicians prescribed longer and more potent opioid prescriptions to replace nonpharmacological therapy during the pandemic [27]. Consequently, the numbers and rates of opioid overdose-related emergency department visits increased by 10.5% and 28.5%, respectively, from 2019 to 2020, despite decreases in visits for other medical emergencies [8]. In Taiwan, the COVID-19 outbreak was well contained (only 447 laboratory-confirmed cases before 30 June 2020) through the mixed approaches of border control, enhanced surveillance, and population-based interventions, such as face mask use [19]. Nevertheless, restricted hospital access and exposure concerns limited, but did not disrupt, medical access and multimodal therapeutic alternatives for outpatients in Taiwan in 2020. In the present study, 30% of patients’ daily opioid doses decreased from 2019 to 2020. Pain medicine specialists reported three (2.6%) patient deaths unrelated to opioid overdose, and six (5.1%) patients were lost to follow-up, suggesting the need to investigate the actual effect of the COVID-19 pandemic in subsequent studies.

### 4.3. Opioid Tapering or Discontinuation and Suicide Attempts

The recognition of an opioid crisis in the United Sates in October 2017 signified a public health emergency [28]. Drug overdose deaths fell for the first time in 25 years in 2018 but climbed to record highs in 2019 and 2020, largely because of illicitly manufactured fentanyl [4]. However, overdose deaths due to prescription opioids in the United States have declined from 36% (17,029 of 70,237 drug overdose deaths) in 2017 to 17.9% (16,416 of 91,799 drug overdose deaths) in 2020 [3,4]. In a 360-day follow-up study of patients receiving long-term high-dose opioid therapy, 9.3% of patients discontinued opioid use, and 26.7% had sustained dosage reductions [9]. In the present study, 30% of Taiwanese patients had reductions (median: 60 MMEs) in their daily opioid dose after a mean 531-day follow-up, including one patient who discontinued all opioid use. Although reducing prescription opioid doses may decrease the risks of overdose and opioid use disorder, rapid tapering or discontinuation can increase the risks of death from overdose or suicide among those receiving high-dose (≥90 MMEs/day) or long-term (>1 year) opioid therapy [10,11]. In the present study, the median prepandemic duration of chronic opioid therapy was 87 months. Less than 5% of patients reported frequent use of more opioid medications than prescribed. However, before the pandemic, more than 20% of patients in the decreased-dose and increased-dose groups occasionally visited the emergency department for additional pain medication. According to the Taiwan Cause of Death Statistics report, intentional self-harm or suicide was the 11th most common cause (*n* = 3656, or 15.5 per 100,000 people) in 2020 [29], but this report did not specify the drug responsible for the overdose. Statistical data regarding the prescription opioid epidemic may thus be scarce or underestimate the severity of the crisis in Taiwan. Therefore, further prospective longitudinal surveys and improved official statistics are needed to aid registered patients with CNCP in Taiwan.

### 4.4. Overall Lower Quality of Life

Chronic pain, daily activity limitations, side effects, and resultant socioeconomic factors all contribute to the complexity of low quality of life of outpatients with opioid prescriptions. The WHOQOL-BREF questionnaire is frequently used to evaluate the effectiveness of therapeutic regimens and psychological interventions for chronic diseases [24]. In the present study, the mean total score (including all four domains: physical health, psychological health, social relationships, and environment) was lower (42/80) than those of Taiwanese and Iranian patients with lower back pain (both 53/80) [30], but higher than that of Korean patients with complex regional pain syndrome (33.5/80) [31]. In the present study, 60% of the patients reported constipation as the most troublesome side effect; this rate is higher than that (47%) in a study of Taiwanese patients receiving long-term opioid therapy in 2010 [18]. Nevertheless, only 34% of our patients took laxatives after the outbreak period in 2020. Additionally, opioid therapy duration, use of nonopioid medications, constipation, depression scores, and aberrant behaviors did not differ among the three groups.

### 4.5. Limitations

This study has several limitations. First, the pain medicine specialists followed up their patients after the first wave of the COVID-19 pandemic in 2020. Six (5.1%) of 117 patients were lost to follow-up, without definite knowledge of their whereabouts, suggesting that the effects of the pandemic on care or opioid misuse may be underestimated among this vulnerable population. In addition, the findings of this study of a limited number of total registered patients with CNCP receiving long-term opioids in Taiwan are not generalizable to other countries with opioid crises. Second, we collected data for opioid prescriptions and nonopioid medications only before and after the first wave of the pandemic; monthly details of all prescriptions were not collected, preventing calculation of tapering rates. Third, because of the pandemic, the scheduled follow-up survey on quality of life and nonpharmacological therapy could not be conducted. To explore the factors potentially contributing to opioid dose reduction or escalation and provide more accurate information on chronic pain management in Taiwan, further studies using the aforementioned surveys are necessary.

## 5. Conclusions

This study demonstrated that most of the registered patients with CNCP in Taiwan continually received long-term opioid prescriptions during the COVID-19 outbreak in 2020, with 70% of the patients receiving daily high-dose opioids (≥90 MMEs) after the outbreak. Less fentanyl patch use was observed among the patients with opioid dose tapering.

## Figures and Tables

**Table 1:** Patient data (*n* = 103).

	Decreased MMEs (Greater than 2.5%)	Equal MMEs (±2.5%)	Increased MMEs (Greater than 2.5%)	*p* Value
*n* (%)	31 (30.1%)	27 (26.2%)	45 (43.7%)	
Male, *n* = 63	17 (54.8%)	19 (70.4%)	27 (60.0%)	0.470 ^a^
Female, *n* = 40	14 (45.2%)	8 (29.6%)	18 (40.0%)	
Age, year	54.7 ± 11.9 (33 to 92)	53.0 ± 11.0 (39 to 89)	53.0 ± 14.3 (30 to 94)	0.824 ^b^
Height, cm	164.4 ± 9.7 (146 to 185)	164.2 ± 5.9 (152 to 176)	162.2 ± 15.3 (100 to 185)	0.992 ^c^
Weight, kg	64.7 ± 14.5 (40 to 97)	62.0 ± 13.6 (41 to 99)	61.1 ± 17.1 (30 to 100)	0.593 ^b^
BMI, kg/m^2^	23.9 ± 4.7 (15.4 to 34.4)	23.0 ± 4.9 (14.5 to 34.3)	23.1 ± 5.0 (15.6 to 33.1)	0.726 ^b^
Follow up duration, days	531.3 ± 56.5 (365 to 619)	535.8 ± 55.8 (454 to 637)	528.8 ± 60.8 (357 to 634)	0.881 ^b^
Pain duration, months	148.7 ± 99.7 (14 to 485), 120	117.7 ± 87.8 (12 to 300), 89	190.8 ± 111.4 (24 to 480), 192	0.013 ^b,^*
Opioid therapy duration, months	108.8 ± 82.4 (4 to 300), 87	107.3 ± 88.4(6 to 300), 78	125.4 ± 100.1 (6 to 420), 120	0.640 ^b^
<12 months, *n* = 6 (5.8%)	2 (6.5%)	1 (3.7%)	3 (6.7%)	0.893 ^d^
12 to <36 months, *n* = 14 (13.6%)	3 (9.7%)	5 (18.5%)	6 (13.3%)	
≥36 months, *n* = 83 (80.6%)	26 (83.9%)	21 (77.8%)	36 (80.0%)	
Pain severity in the past week				
Worst, 0–10	8.2 ± 2.0 (3 to 10)	8.4 ± 1.4 (6 to 10)	9.0 ± 1.5 (3 to 10)	0.074 ^b^
Least, 0–10	4.9 ± 2.7 (0 to 10)	4.3 ± 1.9 (1 to 10)	5.0 ± 2.8 (0 to 10)	0.505 ^b^
Average, 0–10	6.4 ± 2.4 (2 to 10)	5.8 ± 2.1 (3 to 10)	6.8 ± 2.3 (2 to 10)	0.186 ^b^
Pain reduction after medication, %	48.1 ± 21.7 (10 to 100)	49.3 ± 14.7 (10 to 80)	45.8 ± 21.7 (0 to 90)	0.700 ^c^
Daily function pain interference in the past week, in average, 0–10	4.3 ± 2.9 (0 to 9.6)	4.2 ± 2.4 (0 to 9.6)	5.4 ± 2.4 (0 to 10)	0.114 ^b^
Marital status				
Married couple, *n* = 47 (45.6%)	19 (61.3%)	15 (55.6%)	13 (28.9%)	0.010 ^a,#^
Single/divorced/widowed, *n* = 56 (54.4%)	12 (38.7%)	12 (44.4%)	32 (71.1%)	
Work status before pandemic				
Employed, *n* = 20 (19.4%)	7 (22.6%)	5 (18.5%)	8 (17.8%)	0.727 ^d^
Housekeeping, *n* = 16 (15.5%)	5 (16.1)	4 (14.8%)	7 (15.6%)	
Retired, *n* = 23 (22.3%)	9 (29.0%)	7 (25.9%)	7 (15.6%)	
Unemployed, *n* = 44 (42.7%)	10 (32.2%)	11 (40.7%)	23 (51.1%)	
Jobless due to pain, *n* = 81 (78.6%)	25 (80.6%)	21 (77.8%)	35 (77.8%)	0.948 ^a^

Data are presented as a number (%) or as the mean ± standard deviation (SD) (range), median. MME, morphine milligram equivalent. ^a^ *p*-values were estimated by a Chi-square test. ^b^ *p*-values were estimated by one-way ANOVA. ^c^ *p*-values were estimated by Kruskal–Wallis one-way ANOVA. ^d^ *p*-values were estimated by Fisher’s exact test. * *p*-values = 0.516 between the decreased and equal MME groups, 0.016 between the equal and increased MME groups, and 0.217 between the decreased and increased MME groups. # *p*-values = 0.658 between the decreased and equal MME groups, 0.025 between the equal and increased MME groups, and 0.005 between the decreased and increased MME groups.

**Table 2:** Opioid prescriptions, daily opioid dose, and nonopioid medications (*n* = 103).

	Decreased MMEs (Greater than 2.5%)	Equal MMEs (±2.5%)	Increased MMEs (Greater than 2.5%)	*p*-Value
*n* (%)	31 (30.1%)	27 (26.2%)	45 (43.7%)	
MMEs, mg/day				
Before pandemic, *n* = 103	231.1 ± 173.4	202.6 ± 153.4	203.4 ± 232.2	0.804 ^a^
206.5 ± 191.1 (8.0 to 1350.0), 150.0	(22.9 to 705.0), 225.0	(34.0 to 608.4), 150.0	(8.0 to 1350.0), 120.0	
During pandemic, *n* = 103	127. 1 ± 136.9	202.9 ± 153.9	284.9 ± 291.2	0.001 ^b^
215.9 ± 229.5 (0.0 to 1470.0), 140.0	(0.0 to 660.0), 81.3	(34.0 to 612.6), 150.0	(28.8 to 1470.0), 182.8	
Difference	−104.1 ± 120.2	0.3 ± 1.1	81.5 ± 109.1	<0.001 ^b^
	(−600.0 to −1.7), −60.0	(−1.3 to 4.2), 0.0	(8.8 to 595.8), 55.7	
Ratio of difference, %	−44.0 ± 28.9	0.1 ± 0.3	84.2 ± 135.6	<0.001 ^b^
	(−100.0 to −5.0), −46.7	(−0.5 to 1.3), 0.0	(3.0 to 800.0), 40.0	
Opioids < 12 months, *n* = 6 (5.8%)	107.5 ± 31.8	60.0	43.5 ± 43.8	0.377 ^b^
Opioids 12 to < 36 months, *n* = 14 (13.6%)	137.8 ± 156.5	190.6 ± 133.0	204.3 ± 202.0	0.501 ^b^
Opioids ≥ 36 months, *n* = 83 (80.6%)	251.4 ± 177.1	212.3 ± 160.9	216.6 ± 243.9	0.406 ^b^
MMEs, mg/day/kg body weight				
Before pandemic	3.6 ± 2.7	3.4 ± 2.4	3.6 ± 4.3	0.942 ^a^
During pandemic	2.1 ± 2.3	3.4 ± 2.5	4.9 ± 4.8	<0.001 ^b^
MMEs ≥ 200 mg/day				
Before pandemic, *n* = 43 (41.7%)	17 (54.8%)	11 (40.7%)	15 (33.3%)	0.173 ^c^
During pandemic, *n* = 38 (36.9%)	6 (19.4%)	11 (40.7%)	21 (46.7%)	0.047 ^c^
MMEs ≥ 100 mg/day				
Before pandemic, *n* = 64 (62.1%)	22 (71.0%)	18 (66.7%)	24 (53.3%)	0.253 ^c^
During pandemic, *n* = 70 (68.0%)	14 (45.2%)	18 (66.7%)	38 (84.4%)	0.001 ^c^
MMEs ≥ 90 mg/day				
Before pandemic, *n* = 69 (67.0%)	22 (71.0%)	19 (70.4%)	28 (62.2%)	0.662 ^c^
During pandemic, *n* = 73 (70.9%)	14 (45.2%)	19 (70.4%)	40 (88.9%)	<0.001 ^c^
Strong opioids during pandemic, *n* = 103				
Oral morphine, *n* = 57 (55.3%)	15 (48.4%)	14 (51.9%)	28 (62.2%)	0.449 ^c^
MMEs of oral morphine, mg/day	155.5 ± 100.8	211.5 ± 171.1	252.6 ± 229.9	0.286 ^a^
Fentanyl patch, *n* = 19 (18.4%)	1 (3.2%)	8 (29.6%)	10 (22.2%)	0.013 ^d^
MMEs of fentanyl patch, mg/day	28.8	202.2 ± 101.6	278.9 ± 222.9	-
Oxycodone, *n* = 30 (29.1%)	8 (25.8%)	6 (22.2%)	16 (35.6%)	0.430 ^c^
MMEs of oxycodone, mg/day	136.8 ± 213.7	63.7 ± 44.5	130.0 ± 143.8	0.623 ^a^
Concomitant adjuvants during pandemic				
Gabapentinoids, *n* = 39 (37.9%)	10 (32.3%)	10 (37.0%)	19 (42.2%)	0.675 ^c^
NSAID, *n* = 8 (7.8%)	3 (9.7%)	2 (7.4%)	3 (6.7%)	0.898 ^d^
Antidepressants, *n* = 20 (19.4%)	6 (19.4%)	4 (14.8%)	10 (22.2%)	0.744 ^c^
SSRIs, *n* = 11 (10.7%)	1 (3.2%)	3 (11.1%)	7 (15.6%)	0.231 ^d^
Tricyclic antidepressant, *n* = 10 (9.7%)	5 (16.1%)	1 (3.7%)	4 (8.9%)	0.331 ^d^
Benzodiazepine before, *n* = 33 (32.0%)	9 (29.0%)	9 (33.3%)	15 (33.3%)	0.912 ^c^

Data are presented as a number (%) or as the mean ± standard deviation (SD) (range), median. MME, morphine milligram equivalent; NSAID, non-steroidal anti-inflammatory drug; SSRI, selective serotonin reuptake inhibitor. ^a^ *p*-values were estimated by one-way ANOVA. ^b^ *p*-values were estimated by Kruskal–Wallis one-way ANOVA. ^c^ *p*-values were estimated by a Chi-square test. ^d^ *p*-values were estimated by Fisher’s exact test.

**Table 3:** Quality of life and depression scores among patients with chronic noncancer pain (*n* = 103).

	Decreased MMEs (Greater than 2.5%)	Equal MMEs (±2.5%)	Increased MMEs (Greater than 2.5%)	*p*-Value
*n* (%)	31 (30.1%)	27 (26.2%)	45 (43.7%)	
WHO Quality of Life score				
Global Quality of Life, 1–5	2.42 ± 0.96	2.78 ± 0.75	2.18 ± 0.86	0.020 ^a^
Global Health, 1–5	2.06 ± 0.93	2.30 ± 0.91	1.89 ± 0.71	0.139 ^a^
Domain 1: Physical, 4–20	9.37 ± 3.19	10.01 ± 2.64	9.02 ± 3.22	0.412 ^a^
Domain 2: Psychological, 4–20	10.22 ± 3.49	10.62 ± 2.21	9.87 ± 3.53	0.607 ^b^
Domain 3: Social, 4–20	11.65 ± 3.21	12.00 ± 2.51	9.74 ± 3.26	0.004 ^a^
Domain 4: Environmental, 4–20	11.50 ± 2.83	11.93 ± 2.76	11.23 ± 3.03	0.619 ^a^
Total score of 4 domains, 16–80	42.75 ± 11.49	44.56 ± 8.67	39.86 ± 11.68	0.193 ^a^
Constipation, *n* = 62 (60.2%)	20 (64.5%)	19 (70.4%)	23 (51.1%)	0.228 ^c^
Laxative before pandemic, *n* = 28 (27.2%)	7 (22.6%)	8 (29.6%)	13 (28.9%)	0.787 ^c^
Laxative during pandemic, *n* = 35 (34.0%)	8 (25.8%)	9 (33.3%)	18 (40.0%)	0.437 ^c^
Depression diagnosis, *n* = 50 (48.5%)	13 (41.9%)	18 (66.7%)	19 (42.2%)	0.090 ^c^
Suicidal ideation				
Always or frequently, *n* = 14 (13.6%)	7 (22.6%)	1 (3.7%)	6 (13.3%)	0.303 ^d^
Sometimes, *n* = 26 (25.2%)	6 (19.4%)	7 (25.9%)	13 (28.9%)	
Seldom or never, *n* = 63 (61.2%)	18 (58.1%)	19 (70.4%)	26 (57.8%)	
Beck Depression Inventory score, 0–63	20.9 ± 15.8	18.2 ± 12.3	24.3 ± 15.8	0.244 ^a^
0–18 (minimal or mild), *n* = 50	15 (48.4%)	16 (59.6%)	19 (42.2%)	0.375 ^c^
19–63 (moderate or severe), *n* = 53	16 (51.6%)	11 (40.7%)	26 (57.8%)	

Data are presented as a number (%) or as the mean ± standard deviation (SD). MME, morphine milligram equivalent. ^a^ *p*-values were estimated by one-way ANOVA. ^b^ *p*-values were estimated by Kruskal-Wallis one-way ANOVA. ^c^ *p*-values were estimated by a Chi-square test. ^d^ *p*-values were estimated by Fisher’s exact test.

**Table 4:** Concerns regarding opioid discontinuation and misuse behaviors among 103 patients with long-term opioid therapy for chronic noncancer pain.

	Decreased MMEs (Greater than 2.5%)	Equal MMEs (±2.5%)	Increased MMEs (Greater than 2.5%)	*p*-Value
*n* (%)	31 (30.1%)	27 (26.2%)	45 (43.7%)	
Afraid of opioid addiction				
Strongly agree or agree, *n* = 33 (32.0%)	7 (22.6%)	8 (29.6%)	18 (40.0%)	0.161 ^a^
Uncertain, *n* = 12 (11.7%)	2 (7.4%)	6 (22.3%)	4 (8.9%)	
Strongly disagree or disagree, *n* = 58 (56.3%)	22 (71.0%)	13 (48.1%)	23 (51.1%)	
Willing to stop opioids				
Strongly agree or agree, *n* = 26 (25.2%)	6 (19.4%)	8 (29.6%)	12 (26.7%)	0.886 ^a^
Uncertain, *n* = 16 (15.5%)	6 (19.3%)	4 (14.8%)	6 (13.3%)	
Strongly disagree or disagree, *n* = 61 (59.2%)	19 (61.3%)	15 (55.6%)	27 (60.0%)	
Taking more pain medication than prescribed				
Always or frequently, *n*= 5 (4.9%)	2 (6.5%)	2 (7.4%)	1 (2.2%)	0.413 ^a^
Sometimes, *n* = 12 (11.7%)	5 (16.1%)	1 (3.7%)	6 (13.4%)	
Seldom or never, *n* = 86 (83.5%)	24 (77.4%)	24 (88.9%)	38 (84.4%)	
Borrowing pain medication from someone else				
Always or frequently, *n* = 0 (0.0%)	0	0	0	1.000 ^a^
Sometimes, *n* = 2 (1.9%)	1 (3.2%)	0	1 (2.2%)	
Seldom or never, *n* = 101 (98.1%)	30 (96.8%)	27 (100.0%)	44 (97.8%)	
Visiting the emergency room for more pain medication				
Always or frequently, *n* = 5 (4.9%)	2 (6.5%)	1 (3.7%)	2 (4.4%)	0.008 ^a,^*
Sometimes, *n* = 20 (19.4%)	7 (22.5%)	0	13 (28.9%)	
Seldom or never, *n* = 78 (75.7%)	22 (71.0%)	26 (96.3%)	30 (66.7%)	

Data are presented as a number (%) or as the mean ± standard deviation (SD). ^a^ *p*-values were estimated by Fisher’s exact test. * *p*-values = 0.010 between the decreased and equal MME groups, 0.002 between the equal and increased MME groups, and 0.720 between the decreased and increased MME groups.

## Data Availability

The data presented in this study are available upon request from the corresponding author.
